# Acupuncture management for the acute frozen shoulder: A case report

**DOI:** 10.1002/ccr3.5055

**Published:** 2021-11-06

**Authors:** Yun Jin Kim

**Affiliations:** ^1^ School of Traditional Chinese Medicine Xiamen University Malaysia Sepang Malaysia

**Keywords:** acupuncture, case report, frozen shoulder

## Abstract

After an acupuncture treatment 24 times, the symptoms are recovered right shoulder both active and passive full range of movement in all directions, and pain‐free. Furthermore, no adverse effects were observed.

## Graphical abstract

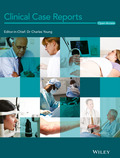

## 1 BACKGROUND

A 45‐year‐old male patient complained about the pain in the right shoulder and restriction of shoulder movement of active and passive motion with abduction and flexion for 5 days. Acupuncture at Zhongping, LI14, and *Ashi* points 24 times on his right shoulder successfully recovered the clinical symptoms.

Frozen shoulder is a common glenohumeral joint condition. The clinical guidelines describe the frozen shoulder as a painful complaint, combined with active and passive glenohumeral range of motion (ROM) restrictions, especially in the external rotation and abduction direction.^1^ This symptom is related to underlying fibrotic processes at the capsuloligamentous structures.^2^ It has been reported that its prevalence ranges from 2% to 5% of the general population, specifically in the age range of 40–60 years old.^3^ The pain can be developed to severe, may cause pronounced sleep disturbance. Restriction of the range of motion is usually more marked with external rotation, but less prominent with the abduction and internal rotation. Although physiotherapy, analgesic, and anti‐inflammatory medications, chiropractic techniques, and exercise therapy are commonly used, the efficacy is variable.^4^ Literature reviews had been implemented in order to evaluation of effectiveness outcomes and pain‐free strength of acupuncture treatment, such as a clinical trial study of Ling Gao et al^5^; they concluded the therapeutic effect of intensive moxibustion plus acupuncture is superior to that of simple acupuncture in improving symptoms of frozen shoulder in patients. In another controlled clinical trial study of Mahsa Asheghan et al^6^, they found out that acupuncture causes improvement of movements of the shoulder in the patients, active and inactive movements in flexion and abduction directions were significantly improved in the case of mentioned movements compared to the past. Therefore, the implementation of acupuncture can be considered as an approach to improve movements of the shoulder in frozen shoulder patients.

## 2 CASE PRESENTATION

We report the case of a 45‐year‐old male university lecturer. The main symptoms of the patient with right shoulder pain, restriction of shoulder movement of both active and passive motion with abduction and flexion for 5 days. He complained of dull and achy pain in the right shoulder and with a sharp pain to the posterior right arm, a hard time computer typing work himself, and unable to do weight training. The patient was experienced similarly medical complain 1 year before and he took the analgesic and anti‐inflammatory medications, after 5 days recovery. The main clinical findings include pain was aggravated by any movement of the right upper limb, lying on the right upper limb and he was awakened during sleep when he rolled onto the affected upper limb. The pain was slightly relieved by taking a hot gel pack on the right shoulder. The main diagnoses are his right glenohumeral joint active ranges of motion (ROM) were as follows: internal rotation 15°, extension rotation 10°, forward flexion 20°, extension 30°, and abduction 10°. The resisted right glenohumeral joint flexion, abduction, and internal and external rotations were graded 3/5. The right glenohumeral joint passive ROM was 5° more in each direction. Posterior and posteroinferior joint play of the right glenohumeral joint was restricted and painful.

## 3 ACUPUNCTURE TREATMENT

**FIGURE 1:**
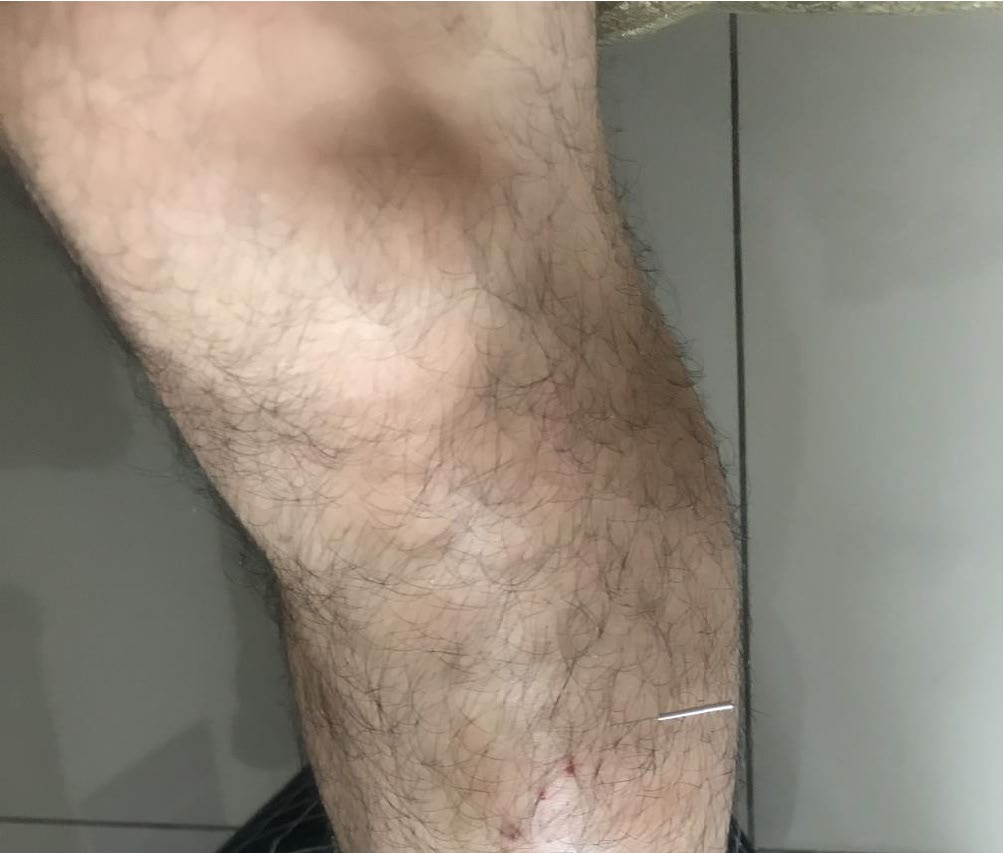
Location of *Zhongping* acupoint

Acupuncture treatment was performed at the extra‐point of *Zhongping* (Left lower limbs), LI 14 (Right), and *Ashi* point (Right locus dolendi points). These acupoints were selected based on the acupuncture point selections on Traditional Chinese Medicine meridian theory to treat frozen shoulder, belongs to “Bi” syndrome, and a published systematic review of acupuncture therapy for scapulohumeral periarthritis.^7^ An extra‐point *Zhongping* is located 1 cun below ST 36^8^ (Figure 1). The patient received acupuncture with a size 0.3 × 45 mm, and 70 mm stainless steel needle (Hansol Medical Co; Reg, no. 141024) was inserted into the muscle layer. Especially, the *Zhongping* point was used deep needling (2.5 *cun*) with the stimulus to *deqi*. The needle was rotated by an experienced acupuncturist with the index finger and thumbs in an alternating clockwise and counter‐clockwise fashion at the rate of three to five rotations per second for additional stimulation every 5 min and a total retention time of 30 min. The acupuncturist returned to check on the patient at regular intervals during the intervention. This treatment was administered four times per week for 6 weeks. All needle placement was performed by an experienced acupuncturist in the School of Traditional Chinese Medicine, Xiamen University Malaysia, and also registered practitioners in the Division of Traditional & Complementary Medicine, Ministry of Health Malaysia. We were informed to the patient about acupuncture in the study as follows: “In this study, acupuncture treatment for frozen shoulder and associated with positive clinical outcomes in clinical studies.”

## 4 CLINICAL OUTCOME

The main outcomes are the patient underwent 3 weeks, the active right glenohumeral abduction, flexion, and external rotation were 35 (10), 60 (20), and 15 (10) degrees, where the passive abduction, flexion, and external rotation were 70, 80, and 20°, respectively. After underwent 6 weeks, the patient had pain‐free and full range of motion (Table 1).

**TABLE 1:** Improvement of the active and passive range of motion (ROM) with acupuncture treatment

ROM		Abduction	Flexion	External rotation
Baseline	Active	10	20	10
	Passive	15	25	15
After 3 weeks	Active	35	60	20
	Passive	70	80	20
After 6 weeks	Active	Full	Full	Full
	Passive	Full	Full	Full

Note

Data showed the degrees.

## 5 DISCUSSION

Frozen shoulder, also known as adhesive capsulitis, is a condition characterized by shoulder stiffness, pain, and limitation of both active and passive range of movement in all directions.^9^ Lundberg suggested patients suffering from frozen shoulder syndrome into “primary” and “secondary.” Primary adhesive capsulitis pertains to those patients who present with no significant findings in the medical history, clinical examination, or medical imaging evaluation to explain their motion limitation and pain, and secondary with a known etiologic factors.^10^ The main symptoms of a patient found usually indicate a gradual onset of shoulder stiffness and pain. The pain is aggravated by the shoulder joint movements, especially external rotation, and sleeping on the involved side, and is relieved by limiting the use of the extremity.^11^ One of long‐term follow‐up analysis of a randomized controlled trial study of Park et al,^12^, the results showed that Bee venom acupuncture and physiotherapy remain clinically effective 1 year after treatment and may help improve long‐term quality of life in patients with adhesive capsulitis of the shoulder. In addition, one of randomized controlled double‐blinded studies of Sven Schröder et al^13^ concluded that acupuncture has a specific impact on adhesive capsulitis beyond the placebo effects that may not only be beneficial in reducing short‐term pain perception, however, may also have a positive clinical outcome for long‐term influence of the time course of recovery. In this case report, we have used the acupoints are *Zhongping*, LI 14, and *Ashi* point. The strengths of the acupuncture management of this case and the implementation of acupuncture cause an improvement in movements of the shoulder in the patient suffering from frozen shoulder. After an acupuncture treatment 24 times, the symptoms are recovered right shoulder both active and passive full range of movement in all directions and pain‐free. Furthermore, no adverse effects were observed. The outcomes in this case report are encouraging and supporting the effectiveness of acupuncture in the management of frozen shoulder. Further studies will needed to enroll large numbers of patients to inform evidence‐based acupuncture practice.

## CONFLICTS OF INTEREST

The author declared no potential conflicts of interest with respect to the research, authorship, and/or publication of this article.

## AUTHOR CONTRIBUTIONS

Yun Jin Kim encountered the case in the TCM Skill Training Clinical Centre took the lead in the management of the patient, and wrote the manuscript.

## ETHICAL APPROVAL

Our institution does not require ethical approval for reporting individual case reports or case series.

## CONSENT

Written informed consent was obtained from the patient for their anonymized information to be published in this case reports article.

## Supporting information

Supplementary Material

Supplementary Material

Supplementary Material

## Data Availability

The clinical data are available in the clinical records of the patient which are stored in the record room of TCM Skill Training Clinical Centre, Xiamen University Malaysia, Sepang, Selangor, Malaysia.
