# Supplementary material for: Acupuncture management for the acute frozen shoulder: A case report
Source: Clin Case Rep. 2021 Nov 6;9(11):e05055. doi: 10.1002/ccr3.5055 (PMC8572337; doi:10.1002/ccr3.5055)
Supplement: Supplementary file 1 — Supplementary Material [file CCR3-9-e05055-s002.pdf]

CARE case report guidelines

CARE Checklist of information to include when writing a case report

(cc) BY-NC-ND

Topic	Item	Checklist item description	Reported on Line
Title	1	The diagnosis or intervention of primary focus followed by the words "case report"	Page 1
Key Words	2	2 to 5 key words that identify diagnoses or interventions in this case report, including "case report"	Page 2
Abstract (no references)	3a	Introduction: What is unique about this case and what does it add to the scientific literature?	Pagr 2
	3b	Main symptoms and/or important clinical findings	Page2
	3c	The main diagnoses, therapeutic interventions, and outcomes	Page2
	3d	Conclusion—What is the main "take-away" lesson(s) from this case?	Page2
Introduction	4	One or two paragraphs summarizing why this case is unique (**may include references**)	Page2
Patient Information	5a	De-identified patient specific information.	Page3
	5b	Primary concerns and symptoms of the patient.	Page3
	5c	Medical, family, and psycho-social history including relevant genetic information	Not applicable for genetic
	5d	Relevant past interventions with outcomes	Page3
Clinical Findings	6	Describe significant physical examination (PE) and important clinical findings.	Page3
Timeline	7	Historical and current information from this episode of care organized as a timeline	Page4
Diagnostic Assessment	8a	Diagnostic testing (such as PE, laboratory testing, imaging, surveys).	Page3
	8b	Diagnostic challenges (such as access to testing, financial, or cultural)	Not applicable
	8c	Diagnosis (including other diagnoses considered)	Page3
	8d	Prognosis (such as staging in oncology) where applicable	Not applicable
Therapeutic Intervention	9a	Types of therapeutic intervention (such as pharmacologic, surgical, preventive, self-care)	Page3 and Page4
	9b	Administration of therapeutic intervention (such as dosage, strength, duration)	Not applicable
	9c	Changes in therapeutic intervention (with rationale)	Page4
Follow-up and Outcomes	10a	Clinician and patient-assessed outcomes (if available)	Page4
	10b	Important follow-up diagnostic and other test results	Page4
	10c	Intervention adherence and tolerability (How was this assessed?)	Page4
	10d	Adverse and unanticipated events	Page5
Discussion	11a	A scientific discussion of the strengths AND limitations associated with this case report	Page5
	11b	Discussion of the relevant medical literature **with references**.	page2,3,4 with ref.5, 6,12
	11c	The scientific rationale for any conclusions (including assessment of possible causes)	Page5
	11d	The primary "take-away" lessons of this case report (without references) in a one paragraph conclusion	Page5
Patient Perspective	12	The patient should share their perspective in one to two paragraphs on the treatment(s) they received	Page3
Informed Consent	13	Did the patient give informed consent? Please provide if requested	Yes ☑ No ☐
